# Monocyte-to-Platelet Ratio Shows Modestly Improved Discrimination Over Model for End-Stage Liver Disease (MELD) 3.0 in Predicting 1-Year Mortality in Patients With Chronic Liver Failure and Hepatic Encephalopathy

**DOI:** 10.7759/cureus.113713

**Published:** 2026-07-31

**Authors:** Jing-tao Qin, Xue-ming Zhang, Kai Liu, Yi-shan He, Shu-qin Zheng, Yuan Xue

**Affiliations:** 1 Department of Infectious Diseases, Xuzhou Medical University-Affiliated Changzhou Hospital, Changzhou, CHN

**Keywords:** chronic liver failure, hepatic encephalopathy, model for end-stage liver disease, monocyte-to-platelet ratio, risk score

## Abstract

Background

This study aimed to develop a noninvasive prognostic model to predict 1-year mortality in patients with chronic liver failure (CLF) and hepatic encephalopathy (HE).

Methods

Clinical data from 285 patients with CLF and HE admitted between January 2010 and April 2024 were analyzed. Multivariate logistic regression analysis was performed to identify independent risk factors associated with 1-year mortality.

Results

Among the 285 patients, 99 (34.7%) died within one year of follow-up. Compared with survivors, non-survivors were older (Z = 3.585, P < 0.01) and had significantly higher Model for End-Stage Liver Disease (MELD), MELD sodium (MELD-Na), and MELD 3.0 scores (Z = 2.863, 2.768, and 2.849, respectively; all P < 0.01). Multivariate regression analysis identified age, monocyte-to-platelet ratio (MPR), lymphocyte count, and alanine aminotransferase (ALT) levels as independent predictors of 1-year mortality (all P < 0.01). A novel prognostic model, Model_age_MPR_Lym_ALT, was developed, demonstrating superior discriminatory power compared to Child-Pugh, MELD, MELD-Na, and MELD 3.0 scores (all P < 0.01). Based on an optimal cutoff value of 0.352, patients were stratified into low-risk (<0.352) and high-risk (≥0.352) groups, with 1-year mortality rates of 21.38% (34/159) and 51.59% (65/126), respectively. Additionally, stratification by age (≥65 years vs. <65 years) demonstrated that the novel model outperformed the traditional scores in the older subgroup.

Conclusion

The proposed noninvasive model, incorporating age, MPR, ALT, and lymphocyte count, may serve as a practical and reliable tool for predicting 1-year mortality in patients with chronic liver failure and hepatic encephalopathy. However, given the retrospective, single-center design, these findings lack external validation and require confirmation in prospective multicenter studies.

## Introduction

Hepatic encephalopathy (HE), a neuropsychiatric complication of cirrhosis or liver failure, has a reported 1-year mortality exceeding 15%[1,2]. Recurrent HE, ranging from minimal to overt HE (OHE), affects approximately 20% of patients with liver cirrhosis (LC), significantly impairing quality of life [3,4].

Although liver transplantation remains the most effective treatment for HE, limited accessibility hinders its widespread application. Existing prognostic models lack sufficient accuracy to effectively prioritize liver transplant candidates. Recently, Lim et al. demonstrated that Model for End-Stage Liver Disease (MELD) 3.0 outperforms the Child-Pugh score, Model for End-Stage Liver Disease (MELD), and MELD sodium (MELD-Na) in predicting 3-month LC-related mortality [5]. However, the predictive value of these noninvasive models for long-term outcomes in HE patients remains unclear.

Impaired ammonia metabolism is a key driver of HE development. Disruption of the urea cycle and glutamine synthesis leads to ammonia accumulation, which contributes to altered consciousness. In addition to hyperammonemia, multiple mechanisms-such as systemic inflammation, mitochondrial dysfunction, gut microbiota dysbiosis, and neurotransmission disturbances-are also involved in HE pathogenesis. Given the critical role of ammonia, current treatment strategies focus on enhancing ammonia clearance or reducing its gastrointestinal production [6].

However, in some patients, ammonia levels remain within normal ranges, and the precipitating factors for HE are not readily apparent. Thus, additional indicators reflecting liver function should be explored to improve HE prognosis prediction [7,8]. Beyond hyperammonemia, systemic inflammation and immune dysfunction are also of major concern. Evidence suggests that elevated ammonia levels can contribute to immune dysfunction [6]. A mouse model of HE, established via bile duct ligation, revealed monocyte and neutrophil accumulation in the brain, underscoring the role of inflammation in HE development [9]. Furthermore, modulation of the gut-liver axis using rifaximin in patients with decompensated LC and intestinal infection has been shown to improve liver function and reduce inflammatory cytokine levels [3].

Given the critical role of inflammation in HE pathogenesis, there is increasing interest in evaluating the prognostic value of inflammation-related indices. Previous studies have assessed combinations of inflammatory and lipid markers-such as the neutrophil-to-high-density lipoprotein cholesterol (HDL-C) ratio (NHR), lymphocyte-to-HDL-C ratio (LHR), and monocyte-to-HDL-C ratio (MHR). Shi et al. recently reported that NHR had a comparable area under the receiver operating characteristic curve (AUROC) to MELD in predicting 12-month mortality in patients with hepatitis B virus (HBV)-related LC and overt HE, and that NHR outperformed both LHR and MHR [10].

Moreover, accumulating evidence indicates that systemic inflammation-based markers - including neutrophil-to-platelet ratio (NPR), monocyte-to-lymphocyte ratio (MLR), and neutrophil-to-lymphocyte ratio (NLR) - are associated with mortality in patients with acute-on-chronic liver failure or LC [11-13]. Another study demonstrated that NLR and platelet-to-lymphocyte ratio (PLR), but not lymphocyte-to-monocyte ratio (LMR), could predict mortality in patients with alcohol-related LC [14]. However, the prognostic value of platelet-related inflammatory markers and their combined predictive capacity for 1-year transplant-free mortality in HE remains poorly understood.

This study aimed to (1) evaluate the prognostic value of inflammation-based indices, particularly the monocyte-to-platelet ratio (MPR), in predicting 1-year mortality among patients with chronic liver failure and hepatic encephalopathy, and (2) develop and validate a novel noninvasive prognostic model incorporating MPR, and compare its performance with established scoring systems including MELD, MELD-Na, and MELD 3.0.

## Materials and methods

Patients and primary endpoint

Clinical data were retrospectively collected from 357 patients diagnosed with chronic liver failure (CLF) and HE who were admitted to Xuzhou Medical University-affiliated Changzhou Hospital between January 2010 and April 2024. Diagnoses of CLF and LC were made in accordance with the Chinese Guideline for the Diagnosis and Treatment of Liver Failure (2019 version) [15]. HE was diagnosed in patients exhibiting cognitive impairment and hyperammonemia and graded according to the West Haven Criteria [16,17]. Cerebrovascular accidents and intracranial tumors were excluded using routine cerebral computed tomography at admission. Patients with hematologic disorders, malignancies, or those who developed hepatocellular carcinoma within the first year of follow-up were excluded from the study. Patients with acute-on-chronic liver failure (ACLF) were also excluded from the study [18]. Demographic, clinical, and laboratory data were collected, including etiology, white blood cell count, neutrophils, lymphocytes, monocytes, platelets, alanine transaminase (ALT), aspartate transaminase (AST), total bilirubin (TBil), albumin, international normalized ratio (INR), and creatinine. All data at admission and one-year outcomes were obtained from the electronic medical records system. Patients with missing values were excluded from the analysis.

The MPR, MLR, NLR, NPR, PLR, neutrophil-to-monocyte ratio (NMR), and lymphocyte-to-platelet ratio (LPR) were calculated using peripheral blood cell counts obtained at admission. These ratios were derived from the absolute counts of monocytes, lymphocytes, neutrophils, and platelets (×10⁹ cells/L). Specifically, MPR = monocyte count/platelet count, MLR = monocyte count/lymphocyte count, NLR = neutrophil count/lymphocyte count, NPR = neutrophil count/platelet count, PLR = platelet count/lymphocyte count, NMR = neutrophil count/monocyte count, LPR = lymphocyte count/platelet count.

The protocol was approved by the ethics committee of Xuzhou Medical University Affiliated Changzhou Hospital in accordance with the Declaration of Helsinki (2013) (ethical approval code of CSYEC(2018)016). Considering that clinical data were collected retrospectively and anonymously, informed consent was waived by the ethics committee of our hospital.

Scoring systems

Noninvasive scoring systems, including MELD, MELD-Na, and MELD 3.0, were calculated as previously described [5,19].

$\mathrm{MELD} = 11.2 \ln(\mathrm{INR}) + 9.6 \ln\!\left(\mathrm{Creatinine}\,(\mathrm{mg/dL})\right) + 3.78 \ln\!\left(\mathrm{TBil}\,(\mathrm{mg/dL})\right) + 6.4$

$\mathrm{MELD\mathrm{-}Na} = \mathrm{MELD} + 1.32\left(137 - \mathrm{Na}\,(\mathrm{mmol/L})\right) - 0.033\,\mathrm{MELD}\left(137 - \mathrm{Na}\,(\mathrm{mmol/L})\right)$

$\begin{aligned} \mathrm{MELD\ 3.0} =\;& 1.33\,(\text{if female}) + 4.56 \ln\!\left[\mathrm{TBil}\,(\mathrm{mg/dL})\right] \\ &+ 0.82\left(137 - \mathrm{Na}\,(\mathrm{mmol/L})\right) \\ &- 0.24\left(137 - \mathrm{Na}\,(\mathrm{mmol/L})\right) \ln\!\left[\mathrm{TBil}\,(\mathrm{mg/dL})\right] \\ &+ 9.09 \ln(\mathrm{INR}) \\ &+ 11.14 \ln\!\left[\mathrm{Creatinine}\,(\mathrm{mg/dL})\right] \\ &+ 1.85\left(3.5 - \mathrm{Albumin}\,(\mathrm{g/dL})\right) \\ &- 1.83\left(3.5 - \mathrm{Albumin}\,(\mathrm{g/dL})\right) \ln\!\left[\mathrm{Creatinine}\,(\mathrm{mg/dL})\right] \\ &+ 6. \end{aligned}$

Where TBil = total bilirubin, INR = international normalized ratio, and Na = sodium.

Statistical analysis

Statistical analyses were performed using SPSS software (version 25.0; IBM Corp., Armonk, NY, USA). Continuous variables were expressed as medians with interquartile ranges (IQRs) and compared using the Mann-Whitney U test. Categorical variables were presented as frequencies (percentages) and analyzed using the chi-square test or Fisher’s exact test, as appropriate. Spearman’s rank correlation coefficient was used for bivariate correlation analysis. Multivariate logistic regression was employed to identify independent risk factors for mortality. All candidate variables were first assessed by univariate analysis, and those with a P value < 0.1 were included in the multivariate binary logistic regression model. Model stability was evaluated using bootstrap resampling (1,000 iterations), with 95% confidence intervals calculated accordingly. The predictive performance of scoring systems was evaluated using the AUROC, calculated with MedCalc software (version 15.2.2; MedCalc Software Ltd., Ostend, Belgium). A two-tailed P-value <0.05 was considered statistically significant.

## Results

Patient characteristics

Of the initial 357 patients, 72 were excluded: 14 developed hepatocellular carcinoma within six months of follow-up, 53 were lost to follow-up, and five were diagnosed with acute-on-chronic liver failure. Ultimately, data from 285 patients were included in the analysis. The etiologies included hepatitis B virus (HBV)-related cirrhosis (n=141), hepatitis C virus-related cirrhosis (n=9), alcoholic cirrhosis (n=33), autoimmune hepatitis (n=19), primary biliary cholangitis (n=12), schistosomiasis-related cirrhosis (n=24), and unknown etiologies (n=47).

During the first year of follow-up, 99 (34.7%) patients died. Compared to survivors, non-survivors were older (Z = 3.585, P < 0.01) and had significantly higher levels of ALT, AST, TBil, creatinine, and C-reactive protein (Z = 2.876, 3.033, 2.605, 2.460, 1.638, 2.208, and 2.905; all P < 0.05). Inflammatory indices including NLR, MLR, NPR, and MPR were also significantly higher in non-survivors (Z = 2.494, 2.820, 2.980, and 2.737; all P < 0.05). While Child-Pugh scores did not differ significantly between groups, MELD, MELD-Na, and MELD 3.0 scores were significantly higher in non-survivors (Z = 2.863, 2.768, and 2.849; all P < 0.01) (Table 1).

**Table 1:** Characteristics of patients with hepatic encephalopathy. Comparison was conducted by Mann-Whitney U test (median and IQR) for continuous variables and Chi-square test for categorical values. Abbreviations: HBV, hepatitis B virus; SBP, spontaneous bacterial peritonitis; UGIB, upper gastrointestinal bleeding; HRS, hepatorenal syndrome; ALT, alanine aminotransferase; AST, aspartate aminotransferase; TBil, total bilirubin; INR, international normalized ratio; WBC, white blood cell; NLR, neutrophils to lymphocytes ratio; NMR, neutrophils to monocytes ratio; MLR, monocytes to lymphocytes ratio; NPR, neutrophils to platelets ratio; LPR, lymphocytes to platelets ratio; MPR, monocytes to platelets ratio; PLR,* *platelet-to-lymphocyte ratio;* *MELD, Model for End-Stage Liver Disease; MELD-Na, MELD sodium.

Variables	Survivor (n=186)	Non-survivor (n=99)	Z or χ^2^	P value
Age, years	62.0(52.0-70.0)	67.0(58.0-73.0)	3.585	<0.01
Male, n (%)	120.0(64.5)	58.0(58.6)	0.969	0.33
HBV infection, n(%)	98.0(52.7)	43.0(43.4)	2.213	0.14
Diabetes, n(%)	36(19.4)	21.0(21.2)	0.139	0.71
Hypertension, n(%)	52(28.0)	38(38.4)	3.251	0.07
Ascites, n(%)	39(21.0)	32(32.3)	4.454	0.04
SBP, n(%)	24(12.9)	28(28.3)	10.245	<0.01
UGIB, n(%)	21(11.3)	25(25.3)	9.306	<0.01
HRS, n(%)	21(11.3)	29(29.3)	14.475	<0.01
ALT, (9-50)U/L	24.0(17.0-35.3)	32.9(19.0-53.0)	2.876	<0.01
AST, (15-40)U/L	36.0(25.0-56.3)	44.0(30.0-75.0)	3.033	<0.01
TBil, (0-23)µmol/L	32.8(19.7-53.8)	37.1(23.7-73.3)	1.638	0.10
Albumin, (40-55)g/L	30.2(25.9-34.0)	30.5(26.9-34.8)	0.842	0.40
Creatinine, (44-97)µmol/L	74.3(62.8-91.9)	83.4(67.4-104.3)	2.208	0.03
Serum sodium, (137-147)mmol/L	140.3(136.8-142.7)	138.8(135.3-141.7)	2.011	0.04
INR (0.8-1.5)	1.4(1.2-1.6)	1.4(1.2-1.6)	1.233	0.22
C-reactive protein, (<5.0)mg/ml	3.6(1.1-9.7)	6.9(1.9-14.5)	2.905	<0.01
WBC, (3.5-9.5)×10^9^/L	4.4(3.2-7.1)	4.8(3.3-6.9)	0.657	0.51
Neutrophils, (1.8-6.3)×10^9^/L	2.3(1.6-4.2)	2.9(1.8-4.6)	1.707	0.09
Lymphocytes, (1.1-3.2)×10^9^/L	1.1(0.7-1.7)	1.0(0.6-1.4)	1.807	0.07
Monocytes, (0.1-0.6)×10^9^/L	0.5(0.3-0.8)	0.5(0.4-0.8)	1.008	0.31
Platelets, (125-350)×10^9^/L	90.0(53.0-150.0)	73.0(52.0-120.0)	1.710	0.09
NLR	2.3(1.4-4.2)	2.8(1.9-5.6)	2.494	0.01
NMR	5.5(3.8-8.0)	5.7(3.9-8.1)	0.761	0.45
MLR	0.4(0.3-0.6)	0.5(0.4-0.8)	2.820	<0.01
NPR	3.0(1.6-4.7)	3.9(2.3-5.6)	2.980	<0.01
LPR	1.1(0.7-1.8)	1.2(0.8-1.8)	0.432	0.67
MPR	0.5(0.4-0.8)	0.7(0.4-0.9)	2.737	<0.01
PLR	87.4(56.7-134.1)	83.3(54.3-131.4)	0.431	0.67
Child-Pugh score	8.0(6.0-9.0)	8.0(6.0-9.0)	1.004	0.32
MELD score	10.7(7.0-14.9)	13.7(8.1-17.1)	2.863	<0.01
MELD-Na score	8.1(3.0-14.4)	12.4(4.6-19.1)	2.768	<0.01
MELD 3.0	9.8(4.9-15.4)	13.5(6.1-19.4)	2.849	<0.01

According to the West Haven Criteria, 147 patients were classified as grade II-IV (moderate to coma), and 138 as grade I (mild). Among these, 50 patients (34.0%) in the grade II-IV group died, compared with 49 (35.5%) in the grade I group. Mortality did not differ significantly between the two groups (χ² = 0.07, P = 0.791).

Independent risk factors for 1-year mortality

Univariate logistic regression analysis identified age, ALT, AST, TBil, creatinine, sodium, MPR, NPR, lymphocyte count, and platelet count as factors associated with 1-year mortality. Additionally, the severity of HE was not associated with 1-year mortality (odds ratio (OR) = 1.068, 95% CI: 0.656-1.740, P =0.791). Multivariate analysis revealed that age, ALT, MPR, and lymphocyte count were independent predictors of 1-year mortality (all P < 0.05). Based on these variables, a prognostic model was constructed:

$P_{\mathrm{Age-MPR-Lym-ALT}}(y=1) = \frac{\exp(-3.907 + 0.044 \times \mathrm{age} + 0.926 \times \mathrm{MPR} - 0.366 \times \mathrm{lymphocytes} + 0.005 \times \mathrm{ALT})}{1 + \exp(-3.907 + 0.044 \times \mathrm{age} + 0.926 \times \mathrm{MPR} - 0.366 \times \mathrm{lymphocytes} + 0.005 \times \mathrm{ALT})}$

The risk factors for 1-year mortality in patients with hepatic encephalopathy are presented in Table 2.

**Table 2:** Risk factors for 1-year mortality in patients with hepatic encephalopathy Abbreviations: HBV, hepatitis B virus; ALT, alanine aminotransferase; AST, aspartate aminotransferase; TBil, total bilirubin; NLR, neutrophils to lymphocytes ratio; NMR, neutrophils to monocytes ratio; MLR, monocytes to lymphocytes ratio; NPR, neutrophils to platelets ratio; LPR, lymphocytes to platelets ratio; MPR, monocytes to platelets ratio; PLR, platelet-to-lymphocyte ratio; WBC, white blood cell; CRP, C-reactive protein; INR, international normalized ratio.

Baseline variables	Univariate			Multivariate			
	Odds ratio	95%CI	P	Odds ratio	95%CI	P	
Age, years	1.039	1.017-1.062	<0.01	1.045	1.022-1.070	<0.01	
Male	0.778	0.472-1.283	0.33	-	-	-	
HBV infection	0.690	0.422-1.126	0.14	-	-	-	
Diabetes	0.891	0.487-1.630	0.71	-	-	-	
ALT, (9-50) U/L	1.005	1.001-1.008	0.01	1.005	1.001-1.009	<0.01	
AST, (15-40) U/L	1.005	1.001-1.008	0.01	-	-	-	
TBil, (0-23) µmol/L	1.006	1.000-1.013	0.07	-	-	-	
Creatinine, (44-97) µmol/L	1.006	1.000-1.011	0.04	-	-	-	
Sodium, (137-147) mmol/L	0.953	0.909-0.999	0.05	-	-	-	
NLR	1.020	0.984-1.056	0.28	-	-	-	
NMR	0.999	0.958-1.042	0.98	-	-	-	
MLR	2.118	1.186-3.782	0.01	-	-	-	
NPR	1.047	0.995-1.102	0.08	-	-	-	
LPR	1.027	0.802-1.316	0.83	-	-	-	
MPR	2.024	1.235-3.318	<0.01	2.523	1.449-4.393	<0.01	
PLR	1.000	0.997-1.003	0.94	-	-	-	
WBC, (3.5-9.5) ×10^9^/L	0.996	0.937-1.060	0.91	-	-	-	
Neutrophils, (1.6-6.3) ×10^9^/L	1.012	0.946-1.083	0.72	-	-	-	
Lymphocytes, (1.1-3.2) ×10^9^/L	0.700	0.497-0.985	0.04	0.694	0.499-0.965	0.03	
Monocytes, (0.1-0.6) ×10^9^/L	1.081	0.575-2.033	0.81	-	-	-	
Platelets, (125-350) ×10^9^/L	0.997	0.993-1.000	0.08	-	-	-	
CRP, (<5) mg/ml	1.009	0.998-1.021	0.12	-	-	-	
INR, (0.8-1.5)	1.614	0.903-2.884	0.11	-	-	-	

**Figure 1:**
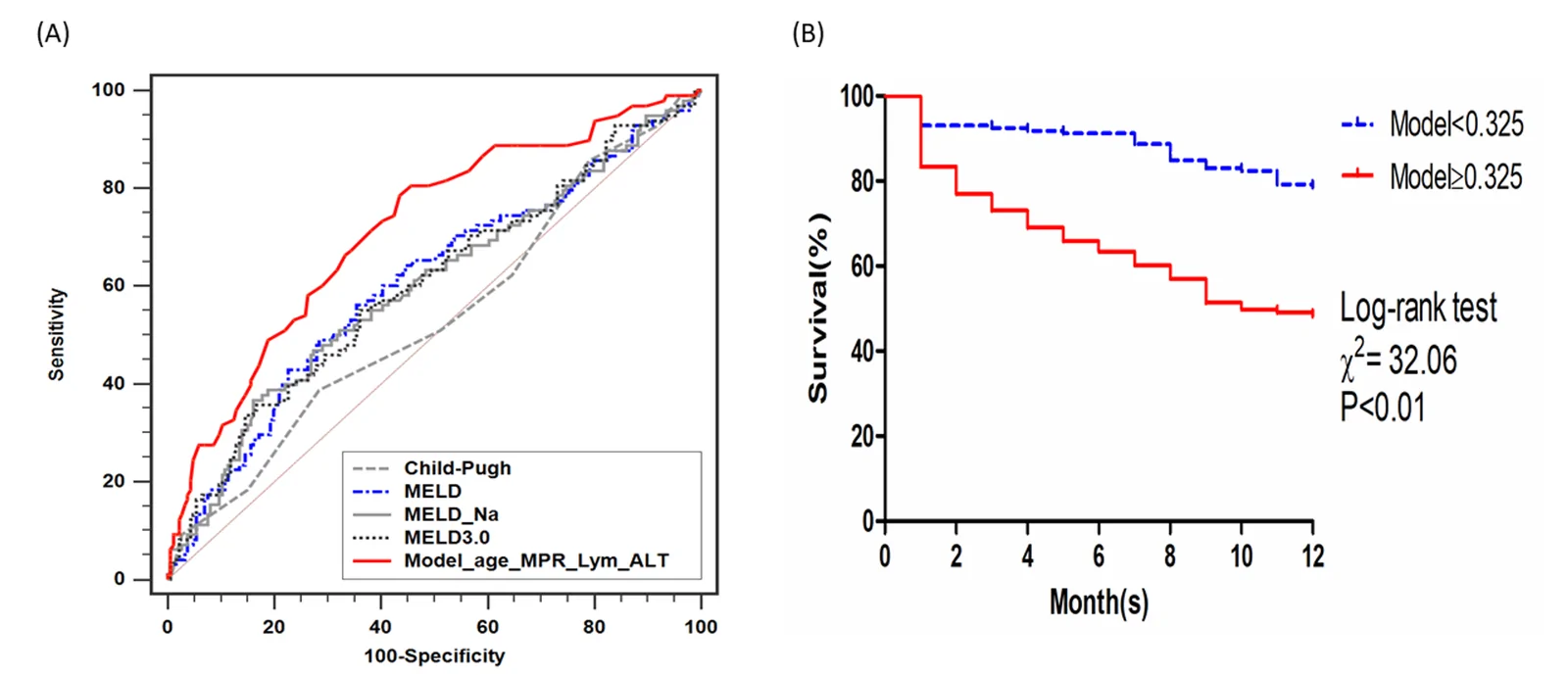
Prognostic evaluation of Model_age_MPR_Lym_ALT. (A) Comparison of AUROC values among Model_age_MPR_Lym_ALT, Child-Pugh, MELD, MELD-Na, and MELD3.0 scores. (B) Kaplan–Meier survival analysis based on Model_age_MPR_Lym_ALT. Abbreviations: AUROC, area under the receiver operating characteristic curve; MPR, monocyte-to-platelet ratio; ALT, alanine transaminase; Model_age_MPR_Lym_ALT, a model consisting of age, MPR, lymphocyte count, and ALT; MELD, Model for End-Stage Liver Disease; MELD-Na, MELD sodium.

The AUROC of the Model_age_MPR_Lym_ALT was 0.716, which was higher than those of the Child-Pugh, MELD, MELD-Na, and MELD3.0 scores (AUROC: 0.535, 0.603, 0.597, and 0.600, respectively; all P < 0.01) (Figure 1A). The model predicted 1-year mortality with a sensitivity of 78.79% and a specificity of 56.45%. Using an optimal baseline cutoff value of 0.352, patients were stratified into two groups: low-risk (<0.352) and high-risk (≥0.352). The 1-year mortality rate was 21.38% (34/159) in the low-risk group and 51.59% (65/126) in the high-risk group. Patients with a Model_age_MPR_Lym_ALT score ≥0.352 had a significantly poorer prognosis (Figure 1B).

Characteristics of patients aged ≥65 years and <65 years

Patients were further stratified by age into older (≥65 years, n=131) and younger (<65 years, n=154) subgroups.

In the older subgroup, non-survivors had significantly higher ALT, AST, and C-reactive protein levels compared to survivors (all P < 0.05) (Table 3). As shown in Table 4, univariate analysis indicated that ALT, AST, MPR, C-reactive protein, and INR were associated with 1-year mortality. Multivariate analysis identified ALT (OR = 1.107, 95% CI: 1.001-1.032, P = 0.04) and MPR (OR = 2.673, 95% CI: 1.094-6.529, P = 0.03) as independent risk factors (Table 4).

**Table 3:** Characteristics of patients with hepatic encephalopathy and age ≥65yrs. Comparison was conducted by Mann-Whitney U test (median and IQR) for continuous variables and Chi-square test for categorical values. Abbreviations: HBV, hepatitis B virus; ALT, alanine aminotransferase; AST, aspartate aminotransferase; TBil, total bilirubin; INR, international normalized ratio; CRP, C-reactive protein; WBC, white blood cell; NLR, neutrophils to lymphocytes ratio; NMR, neutrophils to monocytes ratio; MLR, monocytes to lymphocytes ratio; NPR, neutrophils to platelets ratio; LPR, lymphocytes to platelets ratio; MPR, monocytes to platelets ratio; PLR, platelet-to-lymphocyte ratio; MELD, Model for End-Stage Liver Disease; MELD-Na. MELD sodium.

Variables	Survivor (n=70)	Non-survivor (n=61)	Z or χ^2^	P value
Age, years	72.0(68.8-77.0)	72.0(68.0-75.5)	0.451	0.65
Male, n (%)	37(52.9)	28(45.9)	0.631	0.43
HBV infection, n (%)	22(31.4)	18(29.5)	0.057	0.81
Diabetes, n (%)	17(24.3)	15(24.6)	0.002	0.97
ALT, (9-50) U/L	21.0(13.9-29.0)	30.0(16.9-49.5)	3.394	<0.01
AST, (15-40) U/L	31.0(22.8-43.0)	42.0(29.5-60.5)	2.832	<0.01
TBil, (0-23) µmol/L	29.9(17.9-48.0)	34.7(21.2-63.1)	1.137	0.26
Albumin, (40-55) g/L	30.5(27.1-34.8)	32.1(28.5-35.0)	1.110	0.27
Creatinine, (44-97) µmol/L	76.3(63.7-104.6)	83.4(64.7-108.3)	0.851	0.40
Serum sodium, (137-147) mmol/L	140.9(136.6-143.1)	139.8(136.4-142.3)	1.038	0.30
INR, (0.8-1.5)	1.3(1.1-1.5)	1.3(1.2-1.6)	1.260	0.21
CRP, (<5.0) mg/ml	3.4(1.1-7.7)	7.5(1.7-14.3)	2.494	0.01
WBC, (3.5-9.5) ×10^9^/L	4.3(2.8-7.0)	4.8(3.0-6.8)	0.341	0.73
Neutrophils, (1.8-6.3) ×10^9^/L	2.3(1.5-4.0)	2.9(1.7-4.7)	0.851	0.40
Lymphocytes, (1.1-3.2) ×10^9^/L	0.9(0.6-1.9)	0.9(0.6-1.3)	0.242	0.81
Monocytes, (0.1-0.6) ×10^9^/L	0.4(0.3-0.8)	0.5(0.3-0.7)	0.891	0.37
Platelets, (125-350) ×10^9^/L	89.5(58.5-150.5)	73.0(53.5-123.5)	1.311	0.19
NLR	2.5(1.5-4.7)	2.7(1.9-4.4)	0.856	0.39
NMR	5.6(3.9-8.5)	5.6(4.1-7.9)	0.018	0.99
MLR	0.5(0.4-0.7)	0.5(0.4-0.8)	1.186	0.24
NPR	3.0(1.5-4.2)	3.2(2.1-5.2)	1.647	0.10
LPR	1.0(0.6-1.7)	1.2(0.7-1.8)	0.981	0.33
MPR	0.5(0.3-0.7)	0.6(0.4-0.9)	1.737	0.08
PLR	96.8(58.0-161.5)	84.6(56.9-137.1)	0.981	0.33
Child-Pugh score	7.0(6.0-8.0)	7.0(6.0-9.0)	0.617	0.54
MELD score	10.8(6.4-14.7)	13.0(6.9-17.0)	1.557	0.12
MELD-Na score	7.9(2.2-14.7)	9.8(3.1-18.7)	1.186	0.24
MELD 3.0	8.8(4.9-15.1)	11.9(5.1-19.0)	1.354	0.18

**Table 4:** Risk factors for 1-year mortality in patients with hepatic encephalopathy and age ≥65 years. Abbreviations: HBV, hepatitis B virus; ALT, alanine aminotransferase; AST, aspartate aminotransferase; TBil, total bilirubin; NLR, neutrophils to lymphocytes; NMR, neutrophils to monocytes; MLR, monocytes to lymphocytes; NPR, neutrophils to platelets; LPR, lymphocytes to platelets; MPR, monocytes to platelets; PLR, platelet-to-lymphocyte ratio; WBC, white blood cell; CRP, C-reactive protein; INR, international normalized ratio.

Baseline variables	Univariate			Multivariate		
	Odds ratio	95%CI	P	Odds ratio	95%CI	P
Age, years	0.987	0.927-1.050	0.68	-	-	-
Male	0.757	0.380-1.506	0.43	-	-	-
HBV infection	1.095	0.519-2.310	0.81	-	-	-
Diabetes	1.017	0.457-2.260	0.97	-	-	-
ALT, (9-50) U/L	1.020	1.001-1.039	0.04	1.007	1.001-1.032	0.04
AST, (15-40) U/L	1.014	1.001-1.026	0.03	-	-	-
TBil, (0-23) µmol/L	1.009	0.998-1.020	0.11	-	-	-
Creatinine, (44-97) µmol/L	1.003	0.996-1.009	0.41	-	-	-
Serum sodium, (137-147) mmol/L	0.973	0.915-1.036	0.39	-	-	-
NLR	1.030	0.956-1.109	0.44	-	-	-
NMR	0.974	0.911-1.041	0.44	-	-	-
MLR	1.554	0.734-3.288	0.25	-	-	-
NPR	1.072	0.976-1.177	0.15	-	-	-
LPR	1.179	0.798-1.743	0.41	-	-	-
MPR	2.578	1.084-6.132	0.03	2.673	1.094-6.529	0.03
PLR	0.997	0.992-1.002	0.21	-	-	-
WBC, (3.5-9.5) ×10^9^/L	0.981	0.880-1.094	0.73	-	-	-
Neutrophils, (1.8-6.3) ×10^9^/L	0.995	0.868-1.139	0.94	-	-	-
Lymphocytes, (1.1-3.2) ×10^9^/L	0.780	0.511-1.189	0.25	-	-	-
Monocytes, (0.1-0.6) ×10^9^/L	1.063	0.433-2.608	0.90	-	-	-
Platelets, (125-350) ×10^9^/L	0.997	0.993-1.002	0.27	-	-	-
CRP, (<5.0) mg/ml	1.045	1.004-1.088	0.03	-	-	-
INR (0.8-1.5)	2.432	0.849-6.964	0.09	-	-	-

In the younger subgroup, non-survivors exhibited higher AST, NLR, MLR, NPR, MPR, C-reactive protein, MELD, MELD-Na, and MELD 3.0 scores, and lower sodium levels (P = 0.02) compared to survivors (all other P < 0.05) (Table 5). Univariate analysis showed that ALT, AST, TBil, creatinine, sodium, MLR, and MPR were associated with 1-year mortality. Multivariate analysis identified AST (OR = 1.005, 95% CI: 1.001-1.009, P = 0.02), creatinine (OR = 1.008, 95% CI: 0.999-1.017, P = 0.07), and sodium (OR = 0.917, 95% CI: 0.846-0.994, P = 0.04) as independent predictors (Table 6).

**Table 5:** Characteristics of patients with hepatic encephalopathy and age <65. Comparison was conducted by Mann-Whitney U test (median and IQR) for continuous variables and Chi-square test for categorical values. Abbreviations: HBV, hepatitis B virus; ALT, alanine aminotransferase; AST, aspartate aminotransferase; TBil, total bilirubin; INR, international normalized ratio; CRP, C-reactive protein; WBC, white blood cell; NLR, neutrophils to lymphocytes ratio; NMR, neutrophils to monocytes ratio; MLR, monocytes to lymphocytes ratio; NPR, neutrophils to platelets ratio; LPR, lymphocytes to platelets ratio; MPR, monocytes to platelets ratio; PLR, platelet-to-lymphocyte ratio; MELD, Model for End-Stage Liver Disease; MELD-Na, MELD sodium.

Variables	Survivor (n=116)	Non-survivor (n=38)	Z or χ^2^	P value
Age, years	54.0(46.0-59.8)	56.0(49.0-60.3)	1.106	0.27
Male, n (%)	83.0(71.6)	30.0(78.9)	0.801	0.37
HBV infection, n(%)	76.0(65.5)	25.0(65.8)	0.001	0.98
Diabetes, n(%)	19.0(16.4)	6.0(15.8)	0.007	0.93
ALT, (9-50) U/L	28.0(19.0-47.5)	36.0(25.8-64.6)	1.897	0.06
AST, (15-40) U/L	38.0(27.3-68.5)	54.5(35.0-114.5)	2.567	0.01
TBil, (0-23) µmol/L	35.6(19.8-57.2)	52.2(24.9-88.1)	1.806	0.07
Albumin, (40-55) g/L	29.8(25.1-33.8)	28.5(25.1-33.3)	0.572	0.57
Creatinine, (44-97) µmol/L	71.4(61.0-89.8)	81.2(67.5-97.2)	1.796	0.07
Serum sodium, (137-147) mmol/L	140.2(136.9-142.5)	137.5(134.2-141.1)	2.399	0.02
INR (0.8-1.5)	1.4(1.2-1.6)	1.5(1.3-1.8)	1.337	0.18
CRP, (<5.0) mg/ml	3.9(1.0-11.6)	5.5(2.0-18.0)	1.762	0.08
WBC, (3.5-9.5) ×10^9^/L	4.5(3.3-7.2)	4.7(3.7-7.1)	0.947	0.34
Neutrophils, (1.8-6.3) ×10^9^/L	2.3(1.6-4.2)	3.0(2.0-4.6)	1.781	0.08
Lymphocytes, (1.1-3.2) ×10^9^/L	1.2(0.8-1.6)	1.0(0.6-1.5)	1.419	0.16
Monocytes, (0.1-0.6) ×10^9^/L	0.5(0.3-0.8)	0.6(0.4-0.8)	1.073	0.28
Platelets, (125-350) ×10^9^/L	90.0(52.3-148.8)	75.0(51.8-124)	1.224	0.22
NLR	2.3(1.4-3.7)	3.7(1.9-6.7)	2.699	<0.01
NMR	5.4(3.7-7.9)	6.2(3.3-10.4)	0.895	0.37
MLR	0.4(0.3-0.6)	0.6(0.4-0.9)	2.732	<0.01
NPR	3.0(1.6-4.7)	4.4(2.8-7.3)	2.908	<0.01
LPR	1.2(0.8-1.8)	1.3(0.8-2.0)	0.339	0.73
MPR	0.5(0.4-0.8)	0.7(0.5-1.0)	2.420	0.02
PLR	81.5(54.7-122.0)	79.2(50.9-130.9)	0.339	0.73
Child-Pugh score	8.0(6.0-9.0)	8.0(6.0-10.0)	1.482	0.14
MELD score	10.6(7.1-15.0)	14.6(10.5-19.1)	2.816	<0.01
MELD-Na score	8.4(3.1-14.3)	14.9(8.8-20.4)	3.252	<0.01
MELD3.0	10.2(5.1-15.4)	14.7(10.2-19.9)	3.152	<0.01

**Table 6:** Risk factors for 1-year mortality in patients with hepatic encephalopathy and age <65yrs. Abbreviations: HBV, hepatitis B virus; ALT, alanine aminotransferase; AST, aspartate aminotransferase; TBil, total bilirubin; NLR, neutrophils to lymphocytes ratio; NMR, neutrophils to monocytes ratio; MLR, monocytes to lymphocytes ratio; NPR, neutrophils to platelets ratio; LPR, lymphocytes to platelets ratio; MPR, monocytes to platelets ratio; PLR, platelet-to-lymphocyte ratio; WBC, white blood cell; CRP, C-reactive protein; INR, international normalized ratio.

Baseline variables	Univariate			Multivariate		
	Odds ratio	95%CI	P	Odds ratio	95%CI	P
Age, years	1.029	0.983-1.076	0.22	-	-	-
Male	1.491	0.620-3.587	0.37	-	-	-
HBV infection	0.988	0.457-2.138	0.98	-	-	-
Diabetes	1.045	0.384-2.843	0.93	-	-	-
ALT, (9-50) U/L	1.004	1.000-1.008	0.04	-	-	-
AST, (15-40) U/L	1.005	1.001-1.009	0.02	1.005	1.001-1.009	0.02
TBil, (0-23) µmol/L	1.008	0.999-1.017	0.09	-	-	-
Creatinine, (44-97) µmol/L	1.008	0.999-1.017	0.09	1.008	0.999-1.017	0.07
Serum sodium, (137-147) mmol/L	0.912	0.844-0.987	0.02	0.917	0.846-0.994	0.04
NLR	1.021	0.981-1.063	0.31	-	-	-
NMR	1.025	0.969-1.085	0.39	-	-	-
MLR	3.043	1.282-7.223	0.01	-	-	-
NPR	1.046	0.980-1.118	0.18	-	-	-
LPR	0.994	0.693-1.427	0.97	-	-	-
MPR	2.055	1.067-3.957	0.03	-	-	-
PLR	1.002	0.998-1.006	0.41	-	-	-
WBC, (3.5-9.5) ×10^9^/L	1.020	0.946-1.100	0.61	-	-	-
Neutrophils, (1.8-6.3) ×10^9^/L	1.035	0.955-1.121	0.40	-	-	-
Lymphocytes, (1.1-3.2) ×10^9^/L	0.626	0.349-1.121	0.12	-	-	-
Monocytes, (0.1-0.6) ×10^9^/L	1.233	0.491-3.096	0.66	-	-	-
Platelets, (125-350) ×10^9^/L	0.996	0.991-1.002	0.17	-	-	-
CRP, (<5.0) mg/ml	1.007	0.994-1.020	0.30	-	-	-
INR (0.8-1.5)	1.896	0.881-4.078	0.10	-	-	-

Correlation between MPR and clinical indices in patients aged ≥65 and <65 years

**Figure 2:**
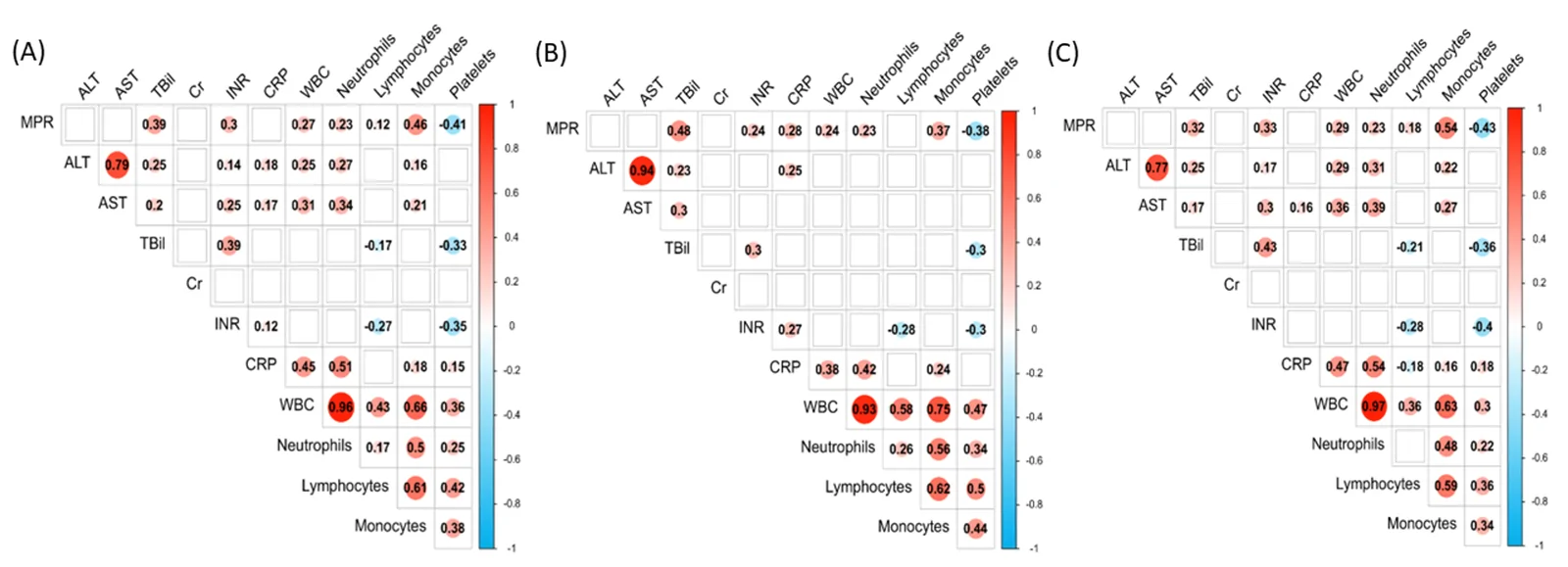
Correlation analysis between MPR and clinical parameters. (A) All patients; (B) Patients aged ≥65 years; (C) Patients aged <65 years. The bubble color indicates the direction of the Spearman's correlation coefficient, with blue representing a negative correlation and red representing a positive correlation. The bubble size is proportional to the absolute value of the correlation coefficient, with larger bubbles indicating stronger correlations. For clarity, the correlation coefficients are annotated only for statistically significant correlations (P > 0.05). The correlation heatmaps were created using a website tool (https://hiplot.com.cn). Abbreviations: MPR, monocyte-to-platelet ratio; ALT, alanine aminotransferase; AST, aspartate aminotransferase; TBil, total bilirubin; INR, international normalized ratio; CRP, C-reactive protein; WBC, white blood cell count; Cr, creatinine.

As illustrated in Figure 2B, in patients aged ≥65 years, MPR was positively correlated with TBil, INR, C-reactive protein, white blood cell count, neutrophil count, and monocyte count (all P < 0.05), and negatively correlated with platelet count (P < 0.05). In patients aged <65 years, MPR was positively correlated with TBil, INR, white blood cell count, neutrophil count, lymphocyte count, and monocyte count (all P < 0.05), and negatively correlated with platelet count (P < 0.05) (Figure 2C).

Comparison of Model_age_MPR_Lym_ALT, Child-Pugh, MELD, MELD-Na, and MELD3.0 in patients aged ≥65 Years and <65 years

**Figure 3:**
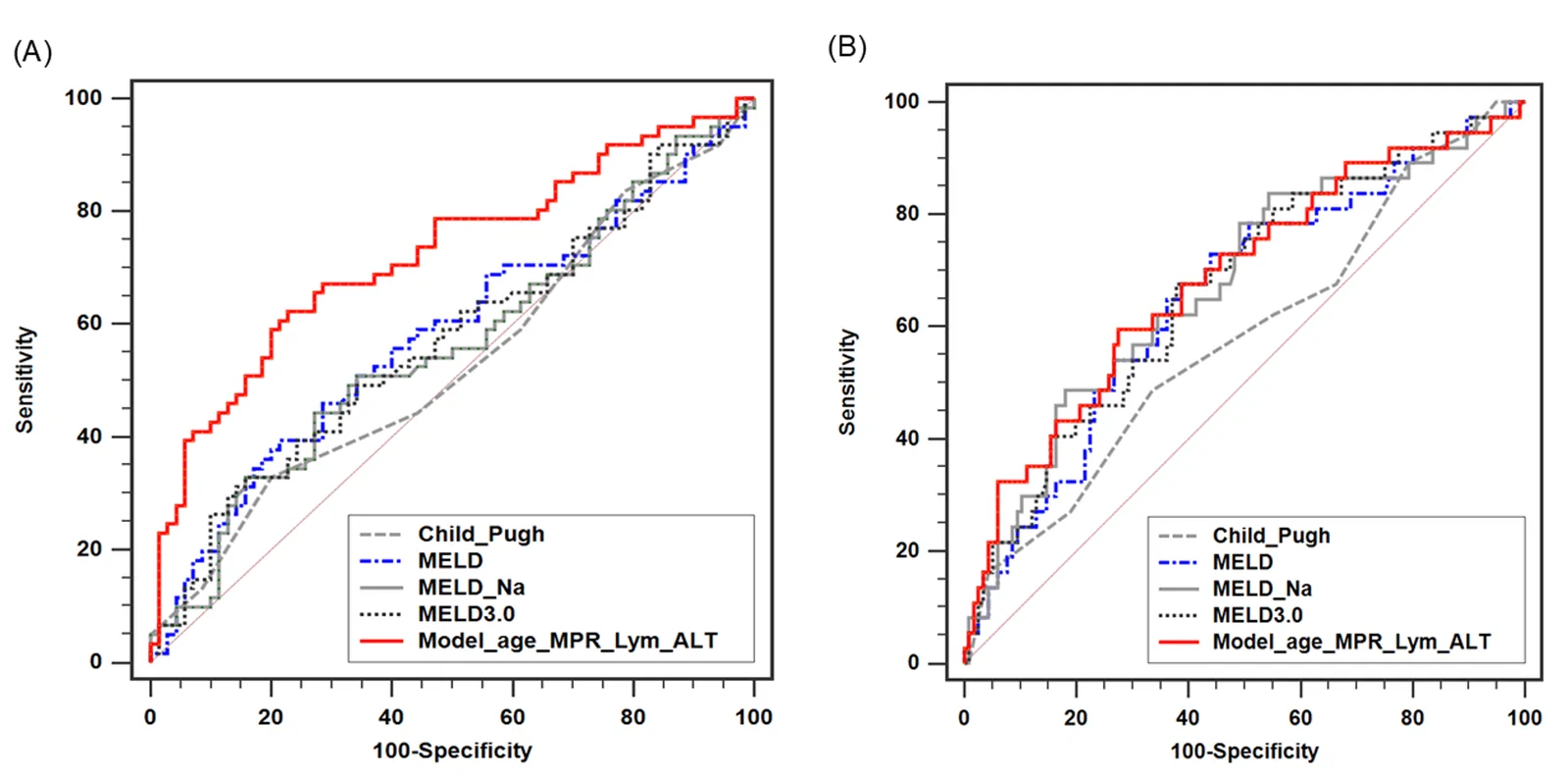
Comparison of AUROC values between Model_age_MPR_Lym_ALT and conventional prognostic models. (A) Patients aged ≥65 years; (B) Patients aged <65 years. Abbreviations: MPR, monocyte-to-platelet ratio; ALT, alanine aminotransferase; Model_age_MPR_Lym_ALT, a model consisting of age, MPR, lymphocyte count, and ALT; MELD, model for end-stage liver disease; MELD-Na, MELD sodium.

Among patients aged ≥65 years, the AUROC of Model_age_MPR_Lym_ALT was 0.722, which was higher than those of the Child-Pugh score, MELD, MELD-Na, and MELD 3.0 (AUROC = 0.531, 0.579, 0.560, and 0.569, respectively; all P < 0.01) (Figure 3A). In patients aged <65 years, Model_age_MPR_Lym_ALT (AUROC = 0.683) demonstrated superiority over the Child-Pugh score (AUROC = 0.580, P = 0.04), but did not outperform the MELD, MELD-Na, or MELD3.0 scores (AUROC = 0.654, 0.672, and 0.666, respectively; all P > 0.05) in predicting 1-year mortality (Figure 3B).

## Discussion

In the present study, 34.7% (99/285) of patients with HE died during the 1-year follow-up period. MELD, MELD-Na, and MELD3.0 scores were significantly higher among non-survivors compared to survivors. Multivariate analysis identified age, ALT, MPR, and lymphocyte count as independent risk factors for 1-year mortality, leading to the development of a novel non-invasive prognostic model, Model_age_MPR_Lym_ALT. Among patients aged ≥65 years, this model (AUROC = 0.716) outperformed the Child-Pugh, MELD, MELD-Na, and MELD3.0 scores. These findings suggest that MPR is an independent prognostic indicator for 1-year mortality in HE and may enhance our understanding of HE prognosis, potentially informing the development of new therapeutic approaches.

Liver transplantation is often not accessible for patients with CLF and HE. Therefore, the identification of simple, cost-effective, and reliable prognostic models is crucial for stratifying patients at high risk of mortality. MPR, derived from routine complete blood counts, has emerged as a promising marker of systemic inflammation and portal hypertension [20,21]. Monocytes, which are precursors of tissue macrophages, reflect systemic inflammation and immune activation [21,22]. Monocytosis is indicative of ongoing innate immune activation [21,22]. In the liver, inflammatory processes recruit and activate monocytes, which then differentiate into pro-inflammatory macrophages, thereby exacerbating hepatic injury and fibrosis. The mechanisms linking monocyte activation to HE progression remain unclear. Platelets are typically reduced in advanced LC due to portal hypertension and hypersplenism [21]. Impaired platelet production and increased consumption may also contribute to CLF progression. In the present study, monocytes were positively correlated with platelet counts. Therefore, the MPR, as a composite ratio, integrates markers of systemic inflammation and portal hypertension, potentially offering superior prognostic sensitivity compared to each parameter alone. In addition, the previously reported inflammatory indices (including NLR and MLR) integrate factors such as inflammation and portal hypertension, but show no significant correlation with the prognosis of HE. The distinction between these conventional indices and the more profound significance of MPR warrants further exploration in future studies.

Accumulating evidence indicates that elevated MPR is associated with increased disease severity across various forms of chronic liver disease [20,23]. In this study, MPR was positively correlated with established indicators of liver disease severity, including TBil and INR, supporting its utility as a prognostic marker. In both older and younger patients, MPR showed positive correlations with WBC, neutrophil count, and lymphocyte count. It cannot be excluded that these elevated systemic inflammatory markers reflect underlying infections secondary to CLF. Given the spectrum of HE manifestations-from covert HE to overt HE and coma-therapies such as non-absorbable antibiotics or probiotics that modulate gut flora and systemic inflammation may be beneficial.

Several non-invasive prognostic models have been evaluated in HE. Calibration assessment was not fully addressed, and our evaluation relied primarily on the AUROC for discriminative performance. In this study, Model_age_MPR_Lym_ALT outperformed the Child-Pugh, MELD, MELD-Na, and MELD3.0 scores. These findings highlight the clinical relevance of age, ALT, monocytes, platelets, and lymphocytes in prognostication. Notably, the model demonstrated superior predictive power only in patients aged ≥65 years. This underscores the clinical utility of incorporating routine hematologic and biochemical parameters into risk assessment.

Among those patients with chronic liver failure, almost half of the patients had HBV infection, and univariate analysis showed that HBV infection was not a significant risk factor for mortality. However, the potential impact of other underlying liver diseases on the prognosis of HE cannot be excluded. Given that different etiologies may overlap-for example, the coexistence of HBV infection and alcohol consumption-further in-depth analysis is challenging. Therefore, no additional etiological analysis was conducted in this study.

This study has several limitations. First, it was a single-center study with a relatively small sample size. Due to the limited sample size, we did not split the data into training and internal validation sets. Instead, we performed subgroup analyses in elderly and non‑elderly patients to explore the model’s predictive performance across different age groups. Large, prospective, multi-center studies are needed to validate these findings. Second, in the present retrospective study, we did not perform a detailed analysis of antibiotic or probiotic regimens due to the considerable heterogeneity in antibiotic types and treatment protocols. It would be of interest to evaluate dynamic changes in MPR or Model_age_MPR_Lym_ALT in patients undergoing treatment with probiotics or microbiota-targeting antibiotics. Third, elucidating the mechanistic link between monocyte activation and HE progression remains an important avenue for future research. For mild HE, the diagnosis relied on mental status changes and hyperammonemia. Owing to the lack of neurophysiological and psychological testing, recurrent versus first-episode HE could not be reliably distinguished, and thus no further analysis was conducted. For precipitating factors, high-protein intake and infection were common. Clinically, high-protein diet-induced HE and hyperammonemia resolved promptly, while infection-induced HE showed worse outcomes, and our findings in the present study also confirmed that patients with coexisting spontaneous bacterial peritonitis (SBP) experienced worse prognosis. Indeed, MPR values may be influenced by a range of factors, including hypersplenism, infection, and transient marrow suppression. Given that SBP may have a confounding effect on MPR values, we opted not to enter infection and MPR into the same multivariate model to avoid potential multicollinearity.

## Conclusions

There remains a lack of optimal risk stratification models for predicting HE prognosis in patients with CLF. The novel non-invasive model, integrating age, MPR, ALT, and lymphocyte count, shows potential as a supplementary prognostic tool and warrants external validation.

## References

[1] Lin L, Huang ZY, Liu K, Tong XC, Zhang ZX, Xue Y (2024). The free triiodothyronine, gamma-glutamyl transpeptidase and spontaneous bacterial peritonitis index: a novel model for predicting 1-year mortality in patients with HBV-related hepatic encephalopathy. Hepat Med.

[2] Bajaj JS, Pompili E, Caraceni P (2025). The burden of hepatic encephalopathy and the use of albumin as a potential treatment. Ann Hepatol.

[3] Sen BK, Pan K, Chakravarty A (2025). Hepatic encephalopathy: current thoughts on pathophysiology and management. Curr Neurol Neurosci Rep.

[4] Häussinger D, Dhiman RK, Felipo V (2022). Hepatic encephalopathy. Nat Rev Dis Primers.

[5] Lim J, Kim JH, Lee A (2025). Predicting mortality and cirrhosis-related complications with MELD3.0: a multicenter cohort analysis. Gut Liver.

[6] Gallego-Durán R, Hadjihambi A, Ampuero J, Rose CF, Jalan R, Romero-Gómez M (2024). Ammonia-induced stress response in liver disease progression and hepatic encephalopathy. Nat Rev Gastroenterol Hepatol.

[7] García-Martínez R, Diaz-Ruiz R, Poncela M (2022). Management of hepatic encephalopathy associated with advanced liver disease. Clin Drug Investig.

[8] Di Cola S, Nardelli S, Ridola L, Gioia S, Riggio O, Merli M (2022). Ammonia and the muscle: an emerging point of view on hepatic encephalopathy. J Clin Med.

[9] Claeys W, Van Hoecke L, Lernout H (2023). Experimental hepatic encephalopathy causes early but sustained glial transcriptional changes. J Neuroinflammation.

[10] Shi K, Wang X, Yi Z, Li Y, Feng Y, Wang X (2025). Inflammatory lipid biomarkers and transplant-free mortality risk in hepatitis B-related cirrhosis and hepatic encephalopathy. Front Med (Lausanne).

[11] Zhang Y, Luo Q, Lin X (2024). Development and validation of a new model including inflammation indexes for the long-term prognosis of hepatitis B-related acute-on-chronic liver failure. J Med Virol.

[12] Zhang N, Wu HS, Pang XQ, Yu CY, Li X, Gao ZL (2025). CD45(+) erythroid progenitor cells as potential biomarkers for disease progression in hepatitis B virus-related acute-on-chronic liver failure. BMC Gastroenterol.

[13] D'Amico T, Miglionico M, Cangemi R (2025). Neutrophil-lymphocyte ratio is associated with worse outcomes in patients with cirrhosis: insights from the PRO-LIVER Registry. Intern Emerg Med.

[14] Dejanović B, Barak O, Čolović P, Janjić N, Savić Ž, Gvozdanović N, Ružić M (2024). Hospital mortality in acute decompensation of alcoholic liver cirrhosis: can novel survival markers outperform traditional ones?. J Clin Med.

[15] Liver Failure and Artificial Liver Group, Chinese Society of Infectious Diseases, Chinese Medical Association; Severe Liver Disease and Artificial Liver Group, Chinese Society of Hepatology, Chinese Medical Association (2019). [Guideline for diagnosis and treatment of liver failure]. Zhonghua Gan Zang Bing Za Zhi.

[16] Xu XY, Ding HG, Li WG (2019). Chinese guidelines on management of hepatic encephalopathy in cirrhosis. World J Gastroenterol.

[17] Blei AT, Córdoba J (2001). Hepatic encephalopathy. Am J Gastroenterol.

[18] Br VK, Sarin SK (2023). Acute-on-chronic liver failure: terminology, mechanisms and management. Clin Mol Hepatol.

[19] Kim WR, Mannalithara A, Heimbach JK (2021). MELD 3.0: the model for end-stage liver disease updated for the modern era. Gastroenterology.

[20] Cai J, Wang K, Han T, Jiang H (2018). Evaluation of prognostic values of inflammation-based makers in patients with HBV-related acute-on-chronic liver failure. Medicine (Baltimore).

[21] Zhou J, Li X, Wang M, Gu C, Liu J (2023). Platelet-to-monocyte ratio as a novel promising agent for the prognosis of hepatitis b virus-associated decompensated cirrhosis. Can J Gastroenterol Hepatol.

[22] Piotrowski D, Sączewska-Piotrowska A, Jaroszewicz J, Boroń-Kaczmarska A (2020). Lymphocyte-to-monocyte ratio as the best simple predictor of bacterial infection in patients with liver cirrhosis. Int J Environ Res Public Health.

[23] Nikiforuk AM, Karim ME, Patrick DM, Jassem AN (2021). Influence of chronic hepatitis C infection on the monocyte-to-platelet ratio: data analysis from the National Health and Nutrition Examination Survey (2009-2016). BMC Public Health.

